# Integrating infectious diseases into life course epidemiology

**DOI:** 10.1093/ije/dyaf059

**Published:** 2025-05-30

**Authors:** Enny S Paixao, Deborah A Lawlor, Mauricio L Barreto, Laura C Rodrigues

**Affiliations:** Department of Infectious Disease Epidemiology and International Health, London School of Hygiene and Tropical Medicine, London, UK; Centro de Integração de Dados e Conhecimentos para Saúde (CIDACS), FIOCRUZ, Salvador, Bahia, Brasil; MRC Integrative Epidemiology Unit, University of Bristol, Bristol, UK; Population Health Science, Bristol Medical School, University of Bristol, Bristol, UK; Centro de Integração de Dados e Conhecimentos para Saúde (CIDACS), FIOCRUZ, Salvador, Bahia, Brasil; Department of Infectious Disease Epidemiology and International Health, London School of Hygiene and Tropical Medicine, London, UK

The term “life course epidemiology” was coined over two decades ago, proposing a framework to investigate the effect of long-term biological, behavioral, and psychosocial processes that link adult health across generations to exposures acting during gestation, childhood, adolescence, and earlier adult life [1, 2]. It is a powerful approach and the recent literature has emphasized the relevance of life course epidemiology in reproductive health [3], women and child health [4], noncommunicable diseases [5], and public health policy prevention strategies [6]. One notable gap in the life course literature is research on the role of infectious diseases—a topic that has often been overlooked, as its importance was highlighted by Hall *et al*. in 2002 [7]. This commentary advocates for including exposure to infectious diseases in life course epidemiology.

## Evidence of the impact of infectious diseases on health across the life course

The impact of prenatal infections on structural and functional development during the intrauterine period, including damage to the fetal nervous system and long-term disabilities, is well documented [8]. For example, congenital infections—particularly those caused by syphilis, toxoplasmosis, cytomegalovirus, and herpes simplex virus (called STORCH agents)—affect offspring globally, leading to clinical manifestations throughout the life course, such as visual impairment, hearing loss, and adverse neurodevelopmental outcomes [8, 9]. The Zika epidemic left thousands of children severely impaired, with disabilities that will remain into adulthood [10]. Similarly, there is evidence of early infectious diseases shaping noninfectious disorders later in life. Some causal links are well established and have influenced policy, such as vaccination programs against human papillomavirus to prevent cervical cancer [11] and against hepatitis B [12] to prevent liver cancer.

Other causal links are under investigation and there is emerging evidence supporting the relationship between infectious diseases experienced in childhood and adulthood and the development of late neurological conditions, including dementia and psychiatric disorders [13]. Furthermore, maternal infections during pregnancy and infectious diseases in childhood have been implicated in the etiology of autism [14], schizophrenia [15], leukemia [16], and type 1 diabetes [17]. Other causal links are biologically plausible and have been discussed, but empirical data are still limited.

## Potential reasons for the neglect of infectious diseases in life course epidemiology

While the evidence of the impact of infections aligns closely with the classical framework of life course epidemiology, infectious diseases are consistently overlooked in the life course literature. This commentary does not aim to provide a comprehensive investigation of this gap. Instead, it offers reflections, including on the history of epidemiology and how it might explain the disconnect between infectious disease and life course epidemiology. One significant factor may be the colonial history of life course theory, developed during what Susser called the Chronic Disease Epidemiology Era. According to Susser, this era, just after World War II, was characterized by the widespread success of antibiotics and vaccines, creating a belief that infectious diseases were no longer a major concern, resulting in a shift in research and public health efforts to understand and prevent noninfectious chronic conditions [18]. Susser himself considered that this shift overlooked the realities of less-developed countries, where chronic infectious diseases such as tuberculosis, syphilis, and malaria were never fully controlled [18]. This historical reason is further compounded by the disproportionate burden of infectious diseases in low- and middle-income countries [19], alongside an imbalance of power in research structures and funding mechanisms, which are often dominated by high-income nations [20], as evidenced by the January 2025 US funding suspensions due to a review of foreign aid policies, which might lead to disruptions to HIV treatment and setbacks in polio eradication efforts [21]. Methodological challenges may also contribute to the lack of inclusion of infectious diseases in life course research. This includes difficulties in obtaining accurate data on who has been infected because of varying symptom severities and the lack of universal testing for most infectious diseases. These challenges were clear during the COVID-19 pandemic [22].

## The need to address this gap and how to approach this

Neglecting infectious diseases in the life course framework limits our understanding of key biological pathways that may be globally significant. There are many gaps in our knowledge of the relationship between infectious diseases and chronic conditions, which could be effectively explored through the lens of life course epidemiology. For instance, it is unclear whether these relationships are pathogen-specific or whether various pathogens can contribute to a spectrum of chronic conditions later in life. Additionally, the potential interactions between infectious and noninfectious exposures, including well-established risk factors such as smoking, and their combined impact on health across the life course remain poorly understood. It is also crucial to comprehend the potential long-term impacts of infection treatments, including antibiotics and vaccines.

Because of the challenge of identifying previous infections and potentially rare adulthood health consequences, large birth cohorts are essential. In particular, these are needed in the Global South, where historically they have been scarce [23]. The recent increase in large birth cohorts in sub-Saharan Africa, South America (notably in Chile) [24], and India [25] alongside large-scale birth cohorts enabled by record linkage studies in Brazil [26] together with new cohorts that are being developed in Pakistan and Sri Lanka [25] are important and need sustained funding to be able to identify the effects of early-life infections and other exposures on long-term health in these countries. Global collaboration is crucial, as research utilizing data from the Global South and underused data on infections—including biomarkers collected across the life course in several high-income countries (e.g. Avon Longitudinal Study of Parents and Children and the Millennium Cohort)—could help to address the knowledge gaps identified here.

## Conclusion

Exploring the role of infectious diseases across the life course, particularly in the development of long-latency conditions, is both timely and critically important. Advancing research in this area has the potential to uncover novel mechanisms underlying disease processes and inform the development of more effective public health interventions, including vaccines, antibiotics, and antiviral therapies. Such efforts could significantly reduce future disease risks, particularly in low- and middle-income countries, where the burden of infectious diseases remains disproportionately high. Nonetheless, the COVID-19 pandemic serves as a stark reminder that infections and their consequences are a global threat, highlighting the international relevance of this research agenda.

## Ethics approval

NA.

## Data Availability

NA.
